# Adenosine and adenosine receptors: Newer therapeutic perspective

**DOI:** 10.4103/0253-7613.55202

**Published:** 2009-06

**Authors:** S. Manjunath, Pranavkumar M. Sakhare

**Affiliations:** Department of Pharmacology, M.R. Medical College, Sedam Road, Gulbarga-585 105, India

**Keywords:** Anaesthesia and critical care, asthma, epilepsy, inflammatory bowel diseases, ischaemia/reperfusion injury, Parkinson's disease, refractory primary pulmonary hypertension

## Abstract

Adenosine, a purine nucleoside has been described as a ‘retaliatory metabolite’ by virtue of its ability to function in an autocrine manner and to modify the activity of a range of cell types, following its extracellular accumulation during cell stress or injury. These effects are largely protective and are triggered by binding of adenosine to any of the four adenosine receptor subtypes namely A1, A2a, A2b, A3, which have been cloned in humans, and are expressed in most of the organs. Each is encoded by a separate gene and has different functions, although overlapping. For instance, both A1 and A2a receptors play a role in regulating myocardial oxygen consumption and coronary blood flow. It is a proven fact that adenosine plays pivotal role in different physiological functions, such as induction of sleep, neuroprotection and protection against oxidative stress. Until now adenosine was used for certain conditions like paroxysmal supraventricular tachycardia (PSVT) and Wolff Parkinson White (WPW) syndrome. Now there is a growing evidence that adenosine receptors could be promising therapeutic targets in a wide range of conditions including cardiac, pulmonary, immunological and inflammatory disorders. After more than three decades of research in medicinal chemistry, a number of selective agonists and antagonists of adenosine receptors have been discovered and some have been clinically evaluated, although none has yet received regulatory approval. So this review focuses mainly on the newer potential role of adenosine and its receptors in different clinical conditions.

## Introduction

**Figure 1:**
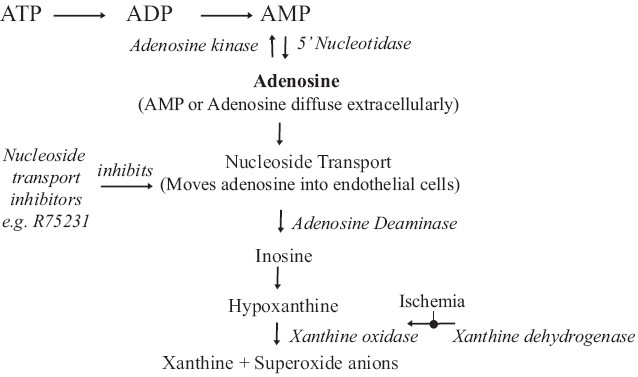
Metabolism of adenosine. ATP, adenosine triphosphate; ADP, adenosine diphosphate; AMP, adenosine monophosphate. Modulating various enzymes/transporters will increase endogenous adenosine concentrations

Adenosine is a metabolite of adenosine triphosphate (ATP), having a very short half-life (1.5 s) due to its rapid metabolism [Figure 1]. It accumulates in the area where ATP is utilised but not reformed, for example during ischaemia and possibly during sepsis. Unlike ATP, adenosine exists free in cytosol of all cells and is transported in and out of the cell by a membrane transporter. It is not a conventional transmitter but a sort of local hormone or better say ‘homeostatic modulator’.

Adenosine is an endogenous purine nucleoside that mediates a wide variety of physiological functions by interacting with four cell surface receptors namely A1, A2a, A2b and A3 [Table 1]. Adenosine is an intermediate metabolite in many important biochemical pathways and has been shown to play a role in the regulation of coronary and systemic vascular tone, platelet function and lipolysis in adipocytes.[1,2] In addition, it mediates important functions like induction of sleep, antioxidant and, antiseizure effects, neuroprotection etc. Until now adenosine was mainly used for terminating paroxysmal supraventricular tachycardia (PSVT) and Wolff Parkinson White (WPW) syndrome. Now, with advances in understanding of adenosine receptors and development of agonists and antagonists [Table 2], adenosine receptors have emerged as potential newer therapeutic targets. Mainly, A2a receptor plays an important role in mediating inflammatory and immune responses.[3] Its actions through the various adenosine receptor subtypes, bring about a decrease in energy demand and an increase in energy supply and thus are protective.[4] Similar to endocannabinoid, the neuromodular adenosine plays a very important integrative role in striatal function.[5] Adenosine and dopamine receptor interactions, also have integrative mechanism in basal ganglia.[6] In addition, several drugs act through modulation of adenosine effect like methylxanthine, dipyridamole, ketamine, beta blockers, calcium channel blockers, dopamine, cannabinoids etc. Adenosine also plays an important role in renal function. Renal tubular sodium transport is the principal consumer of ATP.[7] Sodium transport is influenced by changes in glomerular filtration rate (GFR) and by primary changes in tubular transport. Intrarenal adenosine released by cleavage of ATP, maintains the balance between energy supply and demand by affecting both of these processes. In the renal microcirculation, adenosine receptors exert control over renal blood flow, GFR, renin release and tubuloglomerular feedback.[8]

**Table 1:** Adenosine receptor – Mediated effects in various organ systems

*Receptor subtypes*	*Effects on Stimulating the Receptors*
A_1_	Cardiovascular
	Slows AV nodal conduction (negative dromotropy) ↓ heart rate (negative chronotropy) ↓ atrial contractility (negative inotropy) ↓ β-adrenergic tone Inhibits pacemaker and L-type calcium currents
	Renal
	Inhibits release of renin ↑ reabsorption of sodium in proximal convoluted tubule Vasoconstriction of afferent arteriole - ↓ GFR
	CNS
	↓neurotransmitter release Sedation Anticonvulsant effects
	Metabolic
	Inhibits lipolysis ↑ insulin sensitivity
A2a	Cardiovascular
	Coronary and peripheral vasodilation Inhibits platelet aggregation
A2b	Pulmonary
	Vasodilation Mast cell release of IL-8 → Potential bronchoconstriction and ins ammation
A_3_	Pulmonary
	Mast cell release of allergic mediators → Potential bronchoconstriction

AV indicates atrioventricular, GFR, glomerular filtration rate: IL interleukin

**Table 2:** Comparison of subtypes

			*Adenosine receptors*	

*Receptor*	*Gene*	*Mechanism*	*Agonists*	*Antagonists*
A_1_	ADORA1	G_i/o_ --> cAMP↓ Inhibition ↓ vesicle release, ↓ NMDA receptor activity	N6-Cyclopentyladenosine CCPA, 2'-MeCCPA GR 79236, SDZ WAG 994	Caffeine, Theophylline, 8-Cyclopentyl-1, 3-dimethylxanthine (CPX), 8-Cyclopentyl-1,3-dipropylxanthine (DPCPX), 8-Phenyl 1,3-dipropylxanthine, PSB 36
A_2a_	ADORA2A	G_a_ --> cAMP↑	ATL-146e, CGS-21680, Regadenoson	Caffeine, theophylline, istradefylline, SCH-58261, SCH-442,416 ZM-241,385
A_2b_	ADORA2B	G_s_ --> cAMP↑	5'-N ethylcarboxamidoadenos ne, BAY 60-6583, LUF-5835, LUF-5845.	Theophylline, CVT-6883, MRS-1706, MRS-754, PSB-603, PSB-0788, PSB-1115
A_3_	ADORA3	G_i/o_ --> cAMP↓	2-(1-Hexynyl)-N methyl adenosine, CF-101 (IB-MECA), 2-Cl-IB-MECA, CP-532,903; MRS-3558	Theophylline, MRS-1191, MRS-1220, MRS-1334, MRS-1523, MRS-3777, MRE3008F20, PSB-10, PSB-11, VUF-5574

There is a growing interest in elucidating the mechanisms by which adenosine inhibits inflammation. Hence, these inhibitory adenosine receptors (Gi-A1 and A3) and their downstream signaling pathways are promising targets for newer antiinflammatory therapies. By signalling through the A_2_a adenosine receptors, adenosine suppresses the release of inflammatory mediators,[3] primarily by inhibiting lymphoid or myeloid cells including neutrophils, macrophages, lymphocytes' and platelets.

### Newer potential therapeutic role of adenosine and its receptors

#### Bronchial asthma:

**Figure 2:**
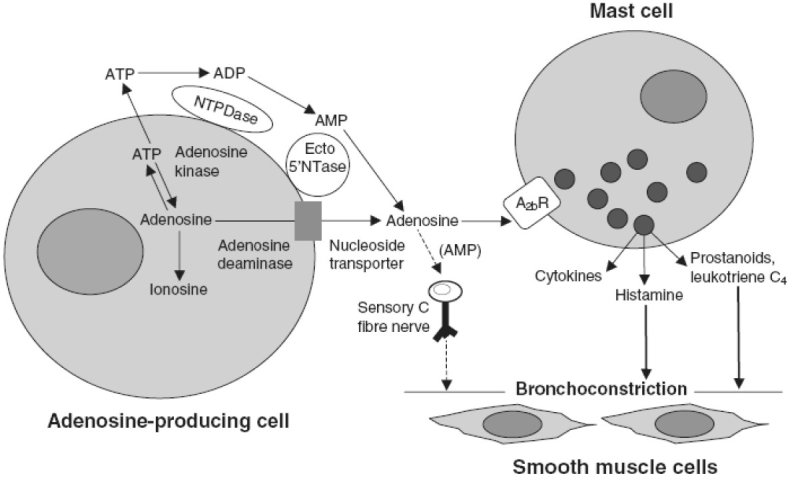
Schematic diagram of the mechanism involved in adenosine-induced bronchoconstriction. Once generated, adenosine activates the adenosine A_2b_ receptors on mast cells. Upon activation of A_2b_ receptors, various inflammatory mediators that induce bronchoconstriction are released. ADP = adenosine diphosphate: AMP = adenosine monophosphate; ATP = adenosine triphosphate; NT = nucleotidase; NTPD= nucleoside triphosphate diphosphohydrolase.

Adenosine, a primordial signalling molecule, produces a number of physiological and pathophysiological effects in the human body. It has been shown that stable form of adenosine, i.e. the nucleotide adenosine monophosphate (AMP) induces bronchoconstriction in asthma, but not in normal airways. Following facts convince that adenosine plays a key role in pathophysiology of asthma and has an important function in acute bronchoconstrictor [Figure 2] and airway inflammatory responses in humans.

- Adenosine levels are increased in broncho-alveolar-lavage fluid[9] and exhaled breath condensate of patients with allergic asthma[10] and in the plasma of patients with exercise-induced asthma.[11]
- The sensitivity of airways to adenosine and adenosine monophosphate (AMP), which is metabolized locally by the 5' nucleotidase to adenosine, more closely reflects an inflammatory process and the phenotype for allergic asthma.[12,13]
- Adenosine induces hyperresponsiveness in the airways of asthmatics, *in vivo* following inhalation and *in vitro* in small airways.[14,15]
- At therapeutic plasma levels, less than those required to inhibit phospho-diesterase enzyme both theophylline, a non-selective adenosine receptor antagonist and bamiphylline, a selective A1 adenosine receptor antagonist (which does not bind to human A_2_b and A_3_ receptors), improve lung function and symptoms in humans with asthma.[16,17]
- Adenosine elicits hyperreactive airway response in humans with allergic asthma by acting on its receptors. All the four adenosine receptors, which have been cloned in humans, are expressed in lung and all are targets for drug development for human asthma.[18]

## Refractory primary pulmonary hypertension (RPPH)

Primary pulmonary hypertension of the newborn (PPHN) is a serious disease in which the pulmonary vascular resistance remains elevated during the neonatal period. It is a clinical syndrome that may occur in association with diverse neonatal cardiorespiratory disorders, such as meconium aspiration, sepsis, pneumonia, acute respiratory distress syndrome, asphyxia, congenital diaphragmatic hernia or lung hypoplasia. As a matter of fact, it is often difficult to rapidly make the correct diagnosis since it may share common pathophysiologic and clinical features with other diseases. Primary pulmonary hypertension of the newborn contributes to neonatal hypoxemia, which is often refractory and remains a major clinical problem, significantly contributing to morbidity and mortality in term and preterm neonates. Indeed, this condition is traditionally treated by correcting the primary and triggering factors, whenever possible and by using conventional protocols including recent ventilation strategies (high-frequency oscillatory ventilation), maintenance of a reasonable electrolytic and acid-base balance, nutritional support and the use of non-specific agents like alkali infusion, magnesium sulphate, prostacyclin, tolazoline and more specific pulmonary vasodilator agents like nitric oxide.[19,20]

Recent reports recommend the use of adenosine infusion for PPHN, alone or associated with other strategies, for refractory scenarios. The pathophysiologic hypothesis is supported by the fact that pulmonary vasodilation is achieved by two known pathways. Nitric oxide acts by elevating intracellular cyclic guanosine monophosphate levels resulting in smooth muscle relaxation with a specific potent vasodilator effect.[21] On the other hand, adenosine causes potent selective pulmonary vasodilation by acting at adenosine receptors (A2) on vascular smooth muscle to increase intracellular cyclic adenosine 3'5' monophosphate (AMP),[22] resulting in smooth muscle relaxation and improvement in systemic and myocardial oxygen delivery. Adenosine may also stimulate K^+^ ATP channels, resulting in hyperpolarization of smooth muscle. The rationale behind its use is more consistent with the fact that patients with pulmonary hypertension have low adenosine levels.

### 

#### Inflammatory bowel diseases (IBDs):

Traditional medical treatments for inflammatory bowel diseases have focused on nonspecific suppression of immune reaction and inflammation with limited efficacy and safety. However, recent advances in the knowledge of enteric immunopathogenesis have paved the way to targeted therapies, allowing a selective blockade of the inflammatory cascade and modulation of key cytokines. In the search for novel therapeutic options, increasing attention is being paid to the adenosine system and its involvement in the pathophysiology of IBDs. The potential therapeutic applications resulting from its pharmacological modulation have been recognized in recent years. The expression of adenosine receptor subtypes in the gastrointestinal tract has been investigated in humans and evidence has been obtained for their localization, both in small and large bowel.

**Figure 3:**
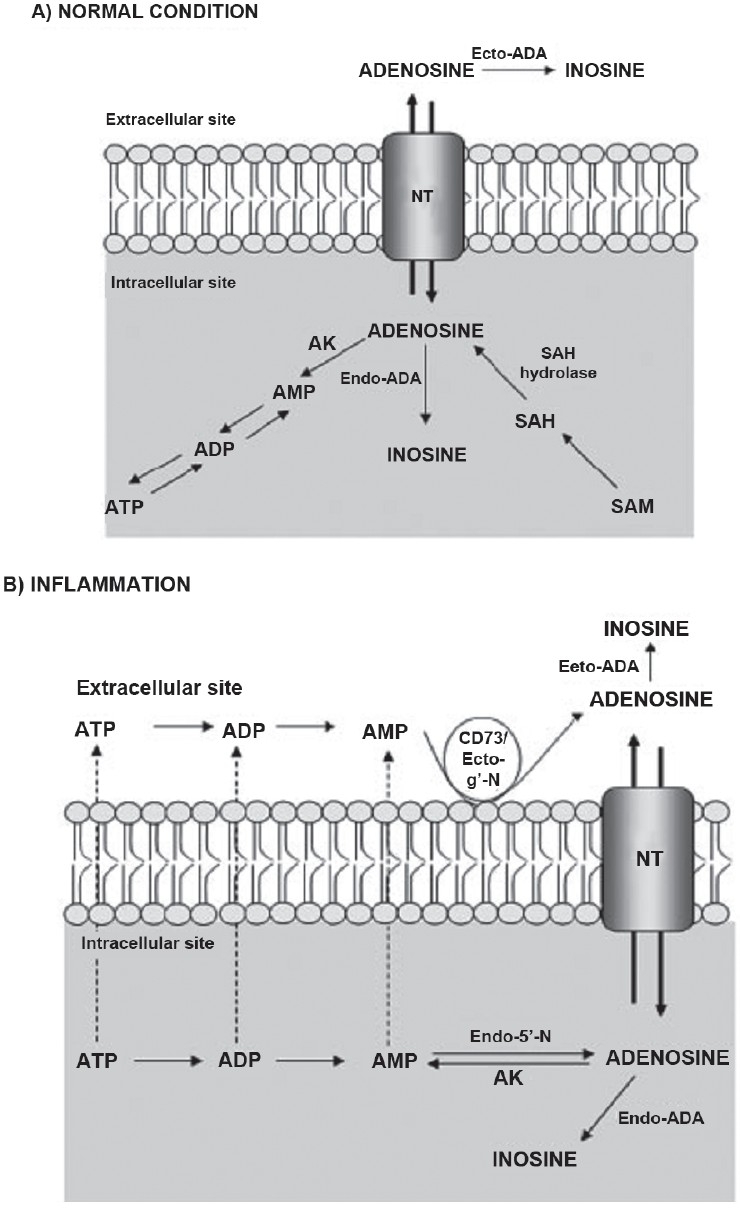
Biosynthesis and catabolism of adenosine under normal conditions (A) or in the presence of inflammation (B). Endo-ADA: endo-adenosine deaminase; Ecto-ADA: ecto-adenosine deaminase; ATP: adenosine triphosphate; ADP: adenosine diphosphate; AMP: adenosine monophosphate; AK: adenosine kinase; SAH: S-adenosylhomocysteine; SAM: S-adenosylmethionine; CD73/Ecto-5-N: ecto-5-nucleotidase; Endo-5-N: endo-5-nucleotidase; NT: nucleoside transporter.

Once released at sites of inflammation, adenosine plays prominent roles in maintaining tissue integrity by modulation of immune functions, down-regulation of phlogistic reactions, interference with the biosynthesis of proinflammatory cytokines and inhibition of neutrophil adhesion, degranulation and anti-oxidant activity.[23] In these settings, the concentrations of adenosine closely reflect the metabolic status of the tissue, and it has been proposed that the purinergic system may act as a sensor apparatus, which provides the immune system with essential information about tissue health, thus contributing to the resolution of inflammation. In gastrointestinal tract, adenosine also contributes to the control of enteric neurotransmission and smooth muscle contractility, thus participating in physiological regulation of gut motor functions. Under physiological conditions, adenosine is mainly formed at the intracellular level from S-adenosylhomocysteine hydrolase. However, in the presence of adverse situations such as hypoxia or inflammation, adenosine production occurs both intracellularly and extracellularly by dephosphorylation of ATP via 5'-nucleotidase enzymes accompanied by suppression of adenosine kinase activity [Figure 3].

As discussed above, adenosine can exert antiinflammatory actions in a variety of systems, including the gastrointestinal tract. The involvement of the adenosine system in the antiinflammatory action has been recognized since the early 1990s. In recent years, increasing interest is being focused on the search for drugs that act via a direct stimulation of adenosine receptor subtypes, in particular A2a and A3 or through an increase in local adenosine concentration and could offer novel therapeutic options for treatment of IBDs. The large body of evidence supporting the prominent role played by A2a receptors in the antiinflammatory actions of adenosine has prompted the synthesis of drugs acting as selective agonists of this receptor subtype and their testing in models of intestinal inflammation. In a study by Odashima *et al*.,[24] the potential antiinflammatory effect of ATL-146e, a selective A2a receptor agonist, was investigated on the acute and chronic model of colitis evoked by formalin-immune complex in rabbits, as well as in a model of spontaneous ileitis in SAMP1/YitFc mice. The stimulation of A2a receptors was associated with a significant amelioration of inflammation in the intestinal mucosa, with a reduction of leucocyte infiltration and inhibition of proinflammatory cytokine levels (TNF-α, IFN-γ and IL-4). However, it has been recently observed that the selective A2a receptor agonist CGS21680 was ineffective in ameliorating various inflammatory parameters of colitis induced by dextran sodium sulphate (DSS) in mice. Overall, the actual significance of A2a receptors in the pathophysiology of intestinal inflammation remains undetermined, and further investigations are required to establish the therapeutic relevance of A2a agonists in IBD. Adenosine A3 receptors are also emerging as possible targets for treatment of bowel inflammation.[25] IB-MECA, an A3 receptor agonist, exerted significant ameliorative effects, both in DSS-induced intestinal inflammation and spontaneous colitis in IL-10 deficient mice. In particular, this drug markedly reduced the colonic levels of the proinflammatory cytokines IL-1, IL-6 and IL-12 and decreased the local production of MIP-1a (Macrophage Inflammatory Protein-1alpha), and MIP-2, with a powerful downregulation of leucocyte trafficking in both models of bowel inflammation. Treatment with IB-MECA prevented the induction of various cytokine/chemokine/inflammatory genes, together with a marked suppression of ROS (Reactive Oxygen Species) production and a significant amelioration of intestinal damage. As an alternative strategy to the direct pharmacological modulation of adenosine receptors, it has been proposed that the elevation of endogenous adenosine concentrations, through the blockade of pivotal catabolic enzymes, might exert a beneficial influence on enteric inflammation. Various authors have reported a significant increase in adenosine-deaminase expression and activity in inflamed tissues, including intestinal ones and the association of a defective adenosine production, with chronicization of the phlogistic conditions.[26] In a model of experimental colitis induced by dinitrobenzene sulphonic acid (DNBS), the results indicated that the blockade of adenosine conversion into inosine, promoted by inhibition of adenosine deaminase, was able to protect the colonic tissues from inflammatory injury. This effect was associated with a significant reduction of TNF-α release, neutrophil infiltration and ROS production.[27] Cellular adenosine uptake is another key event regulating extracellular adenosine concentrations, and therefore the inhibition of nucleoside transporters could represent a further approach to enhance the antiinflammatory actions of this nucleoside. In this regard, a recent *in vitro* study compared the immunomodulatory effects of dipyridamole (a non-selective inhibitor of adenosine uptake) with that of methotrexate, evaluating the effects of these drugs on TNF-α and IL-10 release from intestinal mononuclear cells obtained either from patients with Crohn's disease or healthy controls and stimulated with LPS (lipopolysaccharide) and phytohemagglutinin. The results showed a significant suppression of TNF-α levels in cells treated with both drugs whereas dipyridamole was more effective than methotrexate in increasing IL-10 levels.[28] Thus with development of these adenosine receptor modulators, new door for the treatment of IBD has opened.

#### Anaesthesia and intensive care medicine:

Extracellular adenosine and adenosine triphosphate (ATP) are involved in biological processes including neurotransmission, muscle contraction, vasodilatation, signal transduction and secretion in a variety of cell types.[29] Recently established and potential clinical applications of adenosine, ATP in general and ATP–MgCl2 in intensive care medicine have been well documented.

Several double-blind, placebo-controlled, cross-over studies in healthy human subjects, have shown pain-reducing effects of intravenous adenosine infusion at doses of 50–70 mg/kg/min.[30] In addition, the effectiveness of adenosine in reducing ischaemic pain (70 mg/kg/min IV for 30 min) is comparable to morphine (20 mg/kg/min IV for 5 min) or ketamine (20 mg/kg/min IV for 5 min). Furthermore, adenosine given in combination with morphine or ketamine has an additive effect on pain reduction.[31] A recent study[32] suggested that, adenosine infusion during general anaesthesia for surgery provided good recovery from anaesthesia, associated with pronounced and sustained postoperative pain relief. In this study, adenosine (50–500 mg/kg/min), during surgery-induced pain relief, reduced opioid requirements and attenuated side-effects, such as protracted sedation, cardiorespiratory instability, nausea and vomiting, during the postoperative recovery period. Adenosine was superior to remifentanil (0.05–0.5 mg/kg/min) in all these aspects.

These results suggest that adenosine could be very useful in anaesthesia and intensive care medicine, where it acts by inhibiting nociceptive transmission.

#### Epilepsy:

Current therapies of epilepsy largely rely on the suppression of spontaneous seizures by pharmacotherapy or surgical intervention; however, till date no effective prophylaxis or true pharmacotherapeutic cure is available. Epileptogenesis i.e. the process that leads to epilepsy and spontaneous seizures is thought to be triggered by an initial acute brain injury, e.g. status epilepticus, followed by progressive neuronal cell loss, mossy fibre sprouting and formation of an astrogliotic scar.[33] However, it is presently unclear why some brain injuries evolve into epilepsy while others do not. Therefore, the identification of diagnostic markers to predict epileptogenesis is of utmost importance. The identification of astrogliosis as a hallmark in brain of epileptics, and the identification of astrocytes as important modulators of neuronal activity imply that dysfunction of astrocytes might play a key role in the pathogenesis of epilepsy.[34] Animal models of epileptogenesis have been developed that closely mimic the pathogenesis of human mesial temporal lobe epilepsy, a form of human focal epilepsy that is frequently associated with progression to chronic intractable epilepsy. These models rely on the intrahippocampal,[35] intraamygdaloid or intracerebroventricular administration of small doses of kainic acid (KA). As a primary consequence of KA injection, status epilepticus is elicited, which in turn leads to a characteristic pattern of hippocampal cell death, mossy fibre sprouting, astrogliosis and spontaneous recurrent seizures[35] during a span of several weeks.

Adenosine is an inhibitory modulator of brain activity. By acting on its receptors, mainly by activation of A1 receptors in hippocampus, it exerts predominant inhibitory effects.[36] These inhibitory actions of adenosine can be used therapeutically to suppress seizures[37] and are considered important for maintaining postictal depression[38] and for restoring the metabolic equilibrium following seizures.[39] However, despite more than 20 years of research on the role of adenosine in experimentally induced seizures and the identification of adenosine as endogenous anticonvulsant of the brain,[40] the pathogenic role of the adenosine system in epileptogenesis remains understudied.

Ambient concentrations of adenosine are largely regulated by the activity of its major metabolic enzyme adenosine kinase (ADK),[41] which in the adult brain is predominantly expressed in astrocytes. Consequently, astrogliosis in epilepsy is associated with upregulation of ADK.[42] Transgenic upregulation of ADK in brain leads to a reduction in the tone of ambient adenosine and therefore is associated with increased susceptibility to seizures[43] and ischaemic cell death. These findings provide a neurochemical rationale for therapeutic intervention.[44] Consequently, cultured cells engineered to release adenosine by disrupting their *Adk* gene were tested as therapeutic brain implants in the rat kindling model. These studies have provided the proof-of-concept that focal augmentation of adenosine by cellular brain implants can reduce induced (kindled) seizures and the progression of kindled seizure severity.[45] Hence, adenosine kinase inhibitor like GP515 has been discovered.

However, it remains to be demonstrated whether focal augmentation of adenosine can prevent the development of spontaneous seizures, i.e. true epileptogenesis.

#### Ischaemia/reperfusion (I/R) Injury:

Ischaemic preconditioning (IPC) refers to the mechanism whereby brief periods of ischaemia/reperfusion render a tissue relatively resistant to the harmful effects of subsequent prolonged periods of ischaemia/reperfusion. First described in canine hearts in 1986, IPC has been shown to occur in most species and tissues.[46] The exact mechanism of IPC may vary in different tissues and species where adenosine has an important role.[47–49] This ‘adenosine theory’ is supported by three facts:

- Interstitial adenosine concentration doubles after 5 min of cardiac ischaemia.[50]
- Adenosine antagonists reduce the effect of cardiac IPC.[47,48]
- Adenoreceptor stimulation reduces myocardial damage following ischaemia/reperfusion[51] and during cardiopulmonary bypass.[52]

Adenosine may attenuate ischaemia/reperfusion injury by a number of possible mechanisms,[53] including purine salvaging, improved tissue perfusion, antiinflammatory action and a direct intracellular initiator/effector mechanism.

##### Purine salvaging:

In this process, adenosine acts as a substrate for ATP production. This appears unlikely, as studies have failed to show an improvement in the replenishment of the adenine nucleotide pool.[54]

##### Improved tissue perfusion:

Administration of high doses of adenosine have shown to increase tissue perfusion.[55] Adenosine infusion significantly reduces capillary hyperpermeability, leucocyte adherence and leucocyte extravasation.[56]

##### Antiinflammatory action:

Certain *in vitro* experiments on ischaemia/reperfusion[55] have demonstrated a maximum reduction in granulocyte respiratory burst activity of 20%, using an adenosine concentration of 1 μM. However, the remaining 80% of activity (37 nmol O2/min per million neutrophils) is toxic to cultured endothelial cells.[57] It was concluded that the protective effect observed in the *in vivo* study was due to the 65% decrease in leucocyte extravasation during reperfusion, rather than due to a 20% reduction in respiratory burst activity.[55]

##### Direct intracellular initiator/effector mechanism:

A direct intracellular effect of IPC[53] has been proposed to include all of the preceding observations, while explaining the role of other mediators and the delayed (days or weeks) protective effect of IPC.

By all the above-mentioned mechanisms, adenosine could be very useful in handling the ischaemia/reperfusion injury.

#### Sepsis:

Thiel and colleagues[58] investigated pretreatment of porcine endotoxaemia (*Salmonella abortus equi* endotoxin 5 μg/kg/h IV injection) with adenosine (150 mg/kg/hr intravenous injection started before endotoxin infusion). Adenosine had no effect on endotoxin-induced neutropenia, neutrophil binding/phagocytosis of complement-opsonized zymosan or luminal-enhanced neutrophil chemiluminescence, in response to complement-opsonized zymosan. It did, however, strongly inhibit extracellular superoxide anion release, as measured by lucigenin-enhanced neutrophil chemiluminescence, in response to complement-opsonized zymosan.

Following a soft tissue injury and the induction of hemorrhagic shock,[59] mongrel pigs were resuscitated with Ringer lactate solution (control) or Ringer lactate solution plus acadesine, an adenosine precursor in the dose of (1 or 10 mg bolus intravenously every 12 h, with an initial IV injection of 0.5 mg/kg/min for 30 min). Seventy-two hours later, the animals received *Escherichia coli* 0111:B4 endotoxin (0.5 μg/kg over 30 min). The higher dose acadesine treatment had the following effects:

- Reduced the endotoxin-induced increase in alveolar protein extravasation, systemic oxygen consumption and cardiac index.
- Reduced the endotoxin-induced hypoxia and early transient pulmonary hypertension.
- Reduced the fluid requirement necessary to maintain systemic hemodynamics.
- Reduced mortality and prolonged survival time.

It had no effect on endotoxin-induced leucopoenia or tumour-necrosis factor production. In this study, acadesine may have been acting as an adenosine precursor but also could have been acting as a free radical scavenger,[60] which can be a potential therapeutic tool for treatment of sepsis.

#### Parkinson's disease:

Although current medication treatment of Parkinson's disease (PD) provides good benefit for number of years, long-term treatment remains inadequate. The underlying neuronal degeneration continues to progress and many patients develop long-term complications of the dopamine replacement therapy. Continued neuronal degeneration can lead to the emergence of dementia or imbalance, problems that can cause substantial disability and that are poorly responsive to symptomatic treatment.

Due to these limitations of current therapy, an intense search for new medications to treat PD is ongoing. There is a need for medications that can slow the underlying progression of degeneration, improve PD symptoms in early disease without inducing dyskinesia and improve motor fluctuations and ‘off’ time in advanced disease without worsening dyskinesia. Much interest has focused on non-dopaminergic therapies, especially adenosine A2a receptor antagonists. Istradefylline (KW-6002) is an adenosine A2a receptor antagonist that is now in phase III clinical trials for PD.[61]

Although the anatomy of the A2a receptor distinguishes it from other non-dopaminergic targets in the quest for improved anti-Parkinsonian therapy, it is the behavioural pharmacology of A2a receptor antagonists that has provided the central rationale for their development as anti-Parkinsonian agents. Relatively specific A2a receptor antagonists consistently reverse motor deficits or enhance dopaminergic treatments in animal models of PD. For example, in rats with unilateral 6-hydroxydopamine (6-OHDA) lesions of the dopaminergic pathway, A2a receptor antagonists including KF17837, KW-6002 and MSX-3 potentiated the contralateral turning behaviour induced by levodopa or a dopamine agonist.[62] In addition, motor stimulation by an A2a receptor antagonist in this model showed no tolerance after repeated treatment. Furthermore, the case for developing adenosine A2a receptor antagonists as anti-Parkinsonian therapy has been built on a solid foundation of preclinical evidence.[63]

#### As hypolipidaemic agent:

Coronary artery disease (CAD) is the leading cause of death in the industrialized nations, accounting for 42% of all deaths and for 50% of the total cardiovascular healthcare expenditure.[64] Despite efforts to treat dyslipidaemia with proper diet and drug treatment, CAD remains one of the most common cause of death. Many risk factors (e.g., older age, male gender, hypertension, diabetes, smoking, etc.) are associated with the development and progression of CAD and among them are the serum lipid abnormalities or dyslipidaemias. High total and low density lipoprotein (LDL) cholesterol levels are strongly related to CAD risk, and reductions in LDL levels are associated with reduced coronary disease risk. The importance of elevated triglycerides (TGs) as an independent risk factor of CAD has been controversial. Recently, a metaanalysis of 17 prospective studies of TG levels and cardiovascular disease showed that elevated TG levels can be an independent risk factor for CAD.[65]

RPR749 (molecular formula C22H26F3N7O3, molecular weight 493.49) is a potent and selective adenosine A1 agonist targeted for the management of hypertriglyceridaemia[66] to reduce/normalize TG levels, leading to a reduction in death and morbidity from CAD with little effect on other receptors. The methylated metabolite of RPR749 shows similar pharmacological properties. In the animal models of hypertriglyceridaemia, RPR749 also appears to lower free fatty acid (FFA) and insulin levels and may have additional lipid-modifying effects.

As stated above there is a clear need for developing drugs that can reduce TG levels. The current therapy includes statins, fibrates and niacinic acid. Fibrates (gemfibrozil, fenofibrate and bezafibrate) reduce TGs by approximately 35% and lower LDL at doses of approximately 600 mg/d administered twice daily, but are associated with gallstones and gastrointestinal disorders. Nicotinic acid can also lower TGs by approximately 35% and decrease LDL at doses of 3 g/d given three times daily but is associated with an extremely poor side effect profile and is contraindicated in patients with diabetes. Statins (lovastatin, pravastatin, simvastatin, atorvastatin and cerivastatin) at dose of 20–80 mg/d lower TG levels by approximately 25% and can decrease LDL but may be associated with myopathy. The target was to achieve at least 35% or more lowering of TG levels by RPR749 based on the efficacy of other available therapies. RPR749 (0.1–30 mg/kg) has been shown to reduce TG levels from 20 to 70%[67] and thus could be important tool in treating coronary artery disease.

## Conclusions

Adenosine is very useful therapeutic tool in handling a variety of clinical conditions like ischaemia/reperfusion injury and refractory primary pulmonary hypertension. In addition, it has created its own place in the field of anaesthesia and critical medicine. Alternatives to adenosine administration include modulation of its metabolism and administration of specific agonists/antagonists like:

1. Adenosine precursors (e.g. acadesine).
2. Adenosine analogues—A1 antagonists and A2 agonists both of which are antiinflammatory.
3. Adenosine metabolism inhibitors that increase the concentration of endogenous adenosine. These are:
  - Adenosine deaminase inhibitors (e.g. pentostatin)
  - Adenosine kinase inhibitors (e.g. GP515)
  - Nucleoside transport inhibitors (e.g. R75231). By preventing the uptake of extracellular adenosine into cells, endogenous adenosine is protected from metabolism by adenosine deaminase.

These modulators of adenosine metabolism and specific agonists/antagonists could be very useful in treating certain conditions like bronchial asthma, inflammatory bowel disorders, epilepsy, sepsis etc. Almost all these modulators and agonists/antagonists of adenosine are under clinical trial, but could be potential therapeutic tools.
